# Supplementary material for: The Lipid–Heart Hypothesis and the Keys Equation Defined the Dietary Guidelines but Ignored the Impact of Trans-Fat and High Linoleic Acid Consumption
Source: Nutrients. 2024 May 11;16(10):1447. doi: 10.3390/nu16101447 (PMC11123895; doi:10.3390/nu16101447)
Supplement: Supplementary file 1 [file nutrients-16-01447-s001.zip › nutrients-2981637-supplementary.pdf]

Supplementary Table S1. Composition of selected animal fats and plant-derived oils which were classified as “saturated fat” by Ancel Keys based on their iodine values. These are also referred to as “solid fat” because they all melt above room temperature even though their compositions are very different. Lard is often used to represent saturated fat, although its fatty acid composition and cholesterol content are not representative. Palmitic acid (C16:0) is often used to represent all types of saturated fatty acids, although its physiological properties are not representative of all SFAs. (MCFA: medium-chain fatty acids, C6:0 to C12:0; LCFA: long-chain saturated fatty acids, >C14:0; SFA: saturated fatty acids. (References.: the fatty acid composition and cholesterol content are from FoodData Central: https://fdc.nal.usda.gov/. Iodine values and melting points are from various sources.)

Parameter	Plant-derived oils		Animal fats		
	Coconut oil	Palm oil	Lard	Tallow	Butter
**Fatty acid content, g/100g**					
**C6:0, caproic**	0.48	0.5	0.0	0.0	1.44
**C8:0, caprylic**	6.8	0.0	0.0	0.0	0.84
**C10:0, capric**	5.4	0.0	0.1	0.0	1.92
**C12:0, lauric**	41.8	0.1	0.2	0.9	2.2
**C14:0, myristic**	16.7	1.0	1.3	3.7	7.15
**C16:0, palmitic**	8.64	43.5	23.8	24.9	21.2
**C18:0, stearic**	2.52	4.3	13.5	18.9	7.42
**C18:1, oleic**	6.25	36.6	41.2	36	15
**C18:2, linoleic**	3.36	9.1	10.2	3.1	4.50
**C18:3, linolenic**	0.04	0.2	1.0	0.6	0.639
**Total MCFA**	54.5	0.6	0.3	0.9	6.4
**Total LCFA**	27.9	48.8	38.6	47.5	35.8
**Total SFA**	82.3	49.4	38.9	48.4	42.2
**Total Fatty Acids**	90.5	95.6	95.5	95.6	75.4
**Other parameters**					
**Cholesterol, mg/100g**	0	0	95	109	215
**Iodine value, g I_2/100g**	6 – 11	49 – 55	52 – 68	42 – 48	25 – 42
**Melting point, °C**	25	35	40	50	35

Supplementary Table S2. Descriptions and serum total cholesterol results of the diets used in the National Diet Heart Study (NDHS). The study was conducted at five open centers located in Baltimore, Boston, Chicago, Minneapolis-St. Paul, and Oakland and one closed center at Faribault Hospital in Faribault, Minnesota. Numerous fabricated fat-modified products were developed by the food industry for the various diets. SFA included solid margarine and shortenings, and PUFA included PHO. The participants were instructed to avoid natural saturated fat (coconut oil and animal fat) while *trans*-fats were promoted. SFA = saturated fatty acid; PUFA = polyunsaturated fatty acid; * = not reported. (Reference: National Diet Heart Study, 1968).

Diet	% of Total Calories			PUFA/SFA Ratio	Dietary Cholesterol (mg/day)	Study design	Results
	Total Fat	SFA	PUFA				
**First Study (52-week study)**							
B	30	<9	≥15	1.5	350-450	Fabricated food. Low total fat, low SFA, high PUFA	Lowest TC attained was -33.4 mg/dL from baseline at 2 weeks rising to -24 mg/dL at 52 weeks. Lower total fat of diet B did not lower TC more than the high fat diet C.
C	40	<9	18-20	2.0	350-450	Fabricated food. High total fat, low SFA, high PUFA	Lowest TC attained was -34.1 mg/dL from baseline at 6 weeks and -27.1 mg/dL at 52 weeks. Higher PUFA did not lower TC more than diet B.
D	40	≤18	≤9	0.4	650-750	Fabricated food. High total fat, high SFA, low PUFA, high dietary cholesterol. To represent a high cardiac risk American diet.	Lowest TC attained was -22.6 mg/dL from baseline at 2 weeks and -9.5 mg/dL at 52 weeks. No deaths from heart disease despite high SFA and dietary cholesterol.
X	25-30	*	*	1.0	350	Food purchased by subjects from local grocery. Dietary instructions to eat low total fat and cholesterol.	Average TC level attained was -36.0 mg/dL from baseline at 2 weeks and -29.0 mg/dL at 52 weeks. Similar results to diets B and C despite consumption of local foods with lower total fat.
E	40	*	*	4.4	*	Fabricated food. High total fat, very high PUFA/SFA ratio. Highly controlled diet at the Faribault Hospital.	Lowest TC attained was -46.3 mg/dL from baseline at 6 weeks and -42.1 mg/dL at 52 weeks. TC results do not support a diet with a high PUFA/SFA ratio.
**Extended Study (from week 53 + 11 to 36 weeks using participants from the First Study)**							
BC	30-40	6-7	14-16	1.5-2.0	350-450	Diets B, C and D were combined at three open centers with subgroups who received either provided meat or store-bought meat. Participants were instructed to buy lean meat and trim the fat from the meat.	Average TC attained was -21.0 mg/dL from original baseline after extension, with little difference between subgroups with provided meat and store-bought meat. Consumption of modified meat or low-fat meat did not improve TC results.

F	40	6.5	19	3.0	350-450	High total fat, very low SFA, higher PUFA/SFA ratio than BC diet. Used only store-bought meat.	TC attained was -15.0 to -17.0 mg/dL from original baseline after extension. Higher PUFA did not reduce TC more than Diet BC.
G	40	10	8.7	0.9-1.0	350-450	High total fat, balanced PUFA/SFA ratio. Used only store-bought meat.	At 2 open centers, lowest TC from baseline -5 and -15 mg/dL and -2 and -13 mg/dL after extension. Very similar results to Diet F despite higher SFA and lower PUFA.
B	30	<9	≥15	1.5	350-450	Low total fat, low SFA, high PUFA diet. Continued only at Faribault closed center.	TC from original baseline after 11 more weeks was -40 mg/dL for group on provided meat and -27 mg/dL on store-bought meat. The results were opposite to those of Diet C.
C	40	<9	18-20	2.0	350-450	High total fat, low SFA, high PUFA. Continued only at Faribault closed center.	TC from original baseline after 11 more weeks was -21 mg/dL for group on provided meat and -31 mg/dL on store-bought meat. The results were opposite to those of Diet B.
E	40	*	*	4.4	*	High total fat and very high PUFA/SFA ratio. Highly controlled diet only at Faribault Hospital.	TC from original baseline after 11 more weeks was -41 mg/dL for group on provided meat and -39 mg/dL for group on store-bought meat. Results did not support a low-fat diet.
X	25-30	*	*	1.0	350-450	Food purchased by subject from local grocery. Dietary instructions to eat low total fat and cholesterol.	TC attained was -24.0 mg/dL after extension. Compared to the First Study, the drop in TC was smaller.
**Second Study (28-36 weeks with only new participants)**							
BC	30-40	6-7	14-16	1.5-2.0	350-450	Diets B and C were combined at three open centers with subgroups that received either provided meat or store-bought meat.	TC attained was -25 mg/dL and -26 mg/dL for the two subgroups. Fat consumption from the provided meat and store-bought meat made no difference in TC.
F	40	6.5	19	3.0	350-450	High total fat, low SFA, high PUFA. Used in 2 open centers.	Lowest TC from baseline -27 and -25 mg/dL and -21.0 and -20.0 mg/dL after 36 weeks. Results were similar to Diet BC despite having more PUFA.

G	40	10	8.7	0.9-1.0	350-450	High total fat, balanced PUFA/SFA ratio. Used at 2 open centers.	Lowest TC from baseline -16 and -17 mg/dL and +3 and -12 mg/dL at 36 weeks. Results were similar to Diet D, despite having much less SFA.
D	40	≤18	≤9	0.4	350-450	Fabricated food. High total fat, high SFA, low PUFA, but typical dietary cholesterol. To represent a high cardiac risk American diet in 5 open centers.	The TC results ranged +2 mg/dL to -18 mg/dL but did not increase significantly despite the having very high SFA.
Y	25-30	*	*	1.0	350-450	Food purchased by subject from local grocery. Dietary instructions to eat low total fat and cholesterol.	TC was -24.0 mg/dL at 6 weeks and -22.0 mg/dL at 28 weeks. Compared to Diet X in the First Study, the drop in TC was smaller. No explanation was given.
Z	*	*	*	*	*	No dietary instructions or restrictions. No fabricated food provided. This was also an American diet but was referred to as the true control because, unlike Diet D, it was uncontrolled.	Change in TC of +2 mg/dL from baseline at 36 weeks was not significant. The results of Diet Z showed that a true control group would not significantly change their diet. The results of Diet Z were more representative of the typical American diet than Diet D.

Supplementary Table S3. A brief history of the lipid-heart hypothesis. Please refer to text for details and references.

**1953**	Ancel Keys introduces the lipid-heart hypothesis in presentations and published articles.
**1957**	Keys proposes the first of several equations to try to explain his hypothesis: $$\Delta Chol. = 2.74\ \Delta S - 1.31\ \Delta P$$ where ΔChol. is total serum cholesterol, ΔS is saturated fat, which is conflated with *trans*-fat, and ΔP is polyunsaturated fat, mainly linoleic acid. Keys used hydrogenated coconut oil ("Hydrol") as one of his feeding samples. Using the linear relationship of the square root of iodine values of fat and oil samples with serum cholesterol, Keys defined saturated fat as a fat that raises serum cholesterol.
**1957**	Kummerow and co-workers develop a quantitative method to measure *trans*-fat and report the presence of *trans*-fatty acids in human autopsy and biopsy material. Kummerow also reports that hydrogenated shortenings and margarines contain 23 to 42% *trans* fatty acids.
**1957 - 1966**	The Anti-Coronary Club Study which advocates for reduced consumption of total fat and dietary cholesterol and replacing saturated fat with polyunsaturated fat gives mixed results with fewer heart attacks but more deaths in the Prudent Diet group than the control group, which experienced no deaths.
**1957 - 1984**	The Seven Countries Study (SCS) is a 25-year longitudinal observation study which was organized and lead by Ancel Keys to test the lipid-heart hypothesis. The study concludes that: "Death rates were related positively to average percentage of dietary energy from saturated fatty acids."
**1957**	AHA president Irvine Page receives grants to conduct the National Diet Heart Study (NDHS) of the Prudent Diet using fabricated fat-modified foods. The AHA becomes a fundraising organization and conducts a nationally broadcast telethon.
**1961**	AHA publishes the first advisory on diet identifying intakes of total fat, saturated fat, and cholesterol as the primary causes of heart disease. AHA conflates natural saturated fat with industrial *trans*-fat. Margarine and shortening were widely used at this time and *trans*-fats were unlabeled.
**1963 - 1965**	The National Diet Heart Study (NDHS), which was a 100,000-man study, shows that a low-fat diet does not reduce serum cholesterol levels or cardiac risk more than a high-fat diet and does not prove that replacing saturated fat with polyunsaturated fat reduces deaths from CHD. Funds from the NIH enable the food industry to develop fabricated low-fat processed foods which contain *trans*-fat.
**1965**	Keys publishes a series of four papers which further elaborates on the equation to explain the lipid-heart hypothesis: $$\Delta\ Chol. = 1.2(2\Delta S - \Delta P) + 1.5\Delta Z$$ This equation adds dietary cholesterol, ΔZ, as a contributor to serum cholesterol.
**1967 – 1973**	The Sydney Diet Heart Study is a 6-year study based on the lipid-heart hypothesis involving 458 men. The results do not support the lipid-heart hypothesis. However, the results of this study are not published until 2013.
**1968 - 1973**	The Minnesota Coronary Experiment is a study that was designed and led by Ancel Keys. It involved 9,423 men and women in a five-year study to prove the lipid-heart hypothesis. The results showed that high linoleic acid diets lower serum cholesterol but that those with the largest reduction in serum total cholesterol have the highest incidence of death. The partial results are published in 1989 and a full analysis is published only in 2016.

**1971 - 1980**	The Multiple Risk Factor Intervention (MRFIT) study enlists 12,866 men to test the effects of a low-fat (<35%), low-saturated fat (<8%) diet with high PUFA oils. Total and LDL-C dropped in the test diet group but deaths from cancer were significantly higher for the test diet group (30.6%) compared to the control group (26.5%).
**1973**	Anticipating a change in the diet of Pacific Islanders from the introduction of Western food products, Ian Prior conducts a study on the impact of the high coconut diet on the inhabitants of Pukapuka and Tokelau islands. He published two papers in 1973 and 1981 and reported that "vascular disease is uncommon in both populations and that there is no evidence of the high saturated fat intake having a harmful effect in these populations." Other studies report that coconut oil is not linked to atherosclerosis and heart disease.
**1980**	The first edition of the *Dietary Guidelines for Americans* warns to: "Avoid Too Much Fat, Saturated Fat, and Cholesterol."
**1984**	Keys continues to try to improve his equation but is unable to explain the wide discrepancies in the serum cholesterol results of test subjects in Minnesota which underpredicts serum cholesterol by 5% and the results from test subjects in Massachusetts which overpredicts by 300%. This is his last attempt to try to improve his equation. However, some groups continue to use it in study designs.
**1986**	The results of the Seven Countries Study are published. This study links saturated fat positively to death rates while heart disease is low in groups that consumed olive oil. Polyunsaturated fats do not show any effect. The cohort with the lowest cardiac death rate had one of the highest intakes of total fat.
**1987**	Ancel Keys softens his position on cholesterol in a *New York Times* interview.
**1989**	Ivan Frantz publishes partial results of the Minnesota Coronary Experiment without Keys as co-author.
**1990**	Lipid biochemist Mary Enig, who did her dissertation in 1984 on the effects of *trans*-fat on enzyme systems, reveals the significant under reporting of *trans*-fat in food products and proposes the mandatory labelling of *trans*-fats.
**1992**	Castelli, who was director of the Framingham study from 1979-1995, observes that the more saturated fat and cholesterol one eats, the lower is the serum cholesterol.
**1995**	The International Life Sciences Institute (ILSI), a group that was largely financed by food and chemical corporations, concludes that *trans*-fat does not raise serum cholesterol levels as much as saturated fat and recommend more studies.
**1995**	AHA launches Healthy-Heart label which endorses low-fat products and margarines with *trans*-fats.
**1999**	Analysis of the diet in the SCS reveals that the diet contained margarine.
**2006**	The FDA mandate to label *trans*-fat takes effect. This allows manufacturers to declare "Zero *trans*-fat" if the amount is lower than 0.5 g per serving size.
**2013**	Ramsden and co-workers publish the results of the Sydney Diet Heart Study from recovered raw data. The results show that the PUFA diet lowers serum cholesterol much more effectively compared to control, but the PUFA group had significantly higher rates of all-cause and CVD deaths.
**2015**	*DGA 2015* removes the warning to limit dietary cholesterol to below 300 mg per day, but it continues to warn against dietary cholesterol as part of a healthy eating pattern.
**2015**	FDA revokes GRAS recognition for partially hydrogenated oils (PHO).

**2016**	Ramsden and co-workers publish the results of the Minnesota Coronary Experiment from recovered raw data. The results show that subjects who consumed the PUFA corn oil diet showed reduced serum cholesterol but had a higher incidence of death compared to the control group.
**2017**	The PURE study, which involved 18 high-, medium-, and low-income countries, concludes that high fat intake, including saturated fat, is associated with a lower risk of premature death from all causes, and that saturated fat is not associated with CVD.
**2020**	AHA endorses a dietary pattern that is low in cholesterol.
**2020**	*DGA 2020* continues to support the lipid-heart hypothesis.
